# Photodynamic therapy based on indomethacin-conjugated photosensitizers: targeting cyclooxygenase-2 in cancer treatment

**DOI:** 10.4103/mgr.MEDGASRES-D-25-00210

**Published:** 2026-03-11

**Authors:** Kalayou Hiluf Gebremedhin, Birhanu Kahsay Meresa

**Affiliations:** 1 Department of Chemistry, Collage of Natural and Computational Sciences, Mekelle University, Mekelle, Tigray, Ethiopia; 2 Department of Biotechnology, College of Dryland Agriculture and Natural Resources, Mekelle University, Mekelle, Tigray, Ethiopia

**Keywords:** cancer treatment, cell death, conjugate, cyclooxygenase-2, indomethacin, near infrared, photodynamic therapy, photosensitizer, phototherapy, reactive oxygen species

## Abstract

**Facts**

1. Photodynamic therapy (PDT) kills cancer cells via the generation of reactive oxygen species but can also trigger harmful protumorigenic effects.
2. Cyclooxygenase-2 (COX-2) overexpression and prostaglandin E2 secretion limit PDT efficacy in many tumors.
3. Combining indomethacin (IMC) COX-2 inhibitor with photosensitizer reduces inflammation and enhances PDT treatment.
4. IMC-conjugated photosensitizers show promising targeted, improved tumor accumulation, and effective cancer phototherapy.

**Open Questions**

1. How can PDT-induced protumorigenic inflammation be effectively minimized?
2. Can IMC conjugation with photosensitizers inhibit inflammation and improve the accumulation of photosensitizers in cancer cells?
3. What design strategies best optimize IMC-conjugated photosensitizers?

Photodynamic therapy is a non-invasive treatment that directly kill cancer cells through the generation of reactive oxygen species. However, photodynamic therapy can also paradoxically trigger harmful protumorigenic effects, such as overexpression cyclooxygenase-2 and secretion of prostaglandin E2 in various type of tumors, limiting the overall therapeutic efficacy of photodynamic therapy. The inhibition of prostaglandin E2 secretion using nonselective cyclooxygenase inhibitors such as indomethacin, a widely used anti-inflammatory drug, with photodynamic therapy offers a promising approach for reducing light-induced inflammation and enhancing the effectiveness of photodynamic therapy. In addition, indomethacin and other cyclooxygenase-2 inhibitors have been conjugated with other therapeutic agents to develop tumor-targeted drug delivery systems. Their ability to selectively target and accumulate in cancer cells makes them attractive for delivering a wide range of photosensitizers directly to diseased tissues. Given the considerable progress in this area, this review outlines the photodynamic therapy application of various indomethacin-conjugated photosensitizers for the treatment of cyclooxygenase-2 overexpressed tumor cells, aiming to improve light-mediated effectiveness and reduce off-target effects. Finally, we highlight and discuss future perspectives and challenges in the development of indomethacin-conjugated photodynamic therapy agents.

## Introduction

CCancer is the most frequently diagnosed disease. In 2022, there were about 20 million new cases, 9.7 million deaths, and 53.7 million 5-year prevalent cases.^1^,^2^ This growing burden underscores the urgent need for effective, targeted, and minimally invasive therapies. Among them, photodynamic therapy (PDT) has gained significant attention as a promising alternative and clinically approved cancer treatment.^3^ The overall therapeutic outcomes of PDT depend on three key elements: a photosensitizer (PS; drug or dye), molecular oxygen, and light of a specific wavelength. PDT uses non-toxic PSs that are pharmacologically active only after exposure to a specific wavelength of light in the presence of molecular oxygen. Upon light irradiation, the PS generates reactive oxygen species (ROS) that causes direct cell death and tissue destruction. Compared to systematic drug administration, PDT offers a highly localized and controllable approach, cytotoxic only to areas where light is precisely delivered. Moreover, the PDT therapeutic outcome can be controlled in real-time by adjusting the intensity and duration of light irradiation, allowing for synergistic effects and making PDT particularly advantageous for targeted therapy.^4^ However, a non-specific phototoxicity and photoallergic reaction to healthy tissue remain major challenges that limit the broader application of PDT in clinical use. To address these challenges, several tumor-specific PSs have been reported that selectively accumulate in diseased tissues.^5^ In particular, enzyme-specific PSs that selectively bind to overexpressed tumor-associated enzymes offer great potential to enhance spatial selectivity and therapeutic efficacy of PDT. Thus, this approach ensures that the rational design of tumor-targeted PSs that not only preferentially accumulates in diseased tissue, off-target phototoxicity in health tissues, but also exploits the overexpression patterns of specific molecular targets to achieve improved selectivity.

Among the various molecular targets explored, cyclooxygenase 2 (COX-2) is associated with invasiveness, tumor growth, angiogenesis and metastasis of cancer cells in several solid tumors.^6^ Recent advances have highlighted the development of COX-2-specific fluorescent probes for the detection and imaging of COX-2 expression in a wide range of cancer cells. These probes have been synthesized using non-steroidal anti-inflammatory drugs to enhance tumor-targeting specificity.^7^ Among them, indomethacin (IMC) is frequently used as a preferred non-steroidal anti-inflammatory drug scaffold due to its high binding affinity toward COX-2 enzymes when conjugated to different fluorescent dyes.^8^ These conjugates demonstrated selectivity and tumor targetability. On the other hand, PDT can also paradoxically trigger harmful protumorigenic effects, such as COX-2 overexpression and secretion of prostaglandin E2 (PGE2) in various types of tumors, limiting the overall therapeutic efficacy of PDT. To overcome this, our and other groups have developed IMC-conjugated PSs based on the amide derivative of IMC with various photosensitizing moieties via linker chemistry.^9^,^10^,^11^ These hybrid PDT agents have shown promising potential in PDT for cancer treatment due to their tumor selectivity and enhanced phototoxicity under light irradiation.

This review aims to provide an overview of recent developments of IMC-conjugated PSs, focusing on their design, mechanism of action, and therapeutic performance in COX-2 highly expressed cancer cells.

## Search Strategy

A systematic literature review was analyzed using the Web of Science database to identify studies published up to July 30, 2025, focusing on cancer, COX-2, photosensitizer, PDT, and indomethacin. The search strategy combined keywords, including “cancer AND COX-2 AND photodynamic therapy OR photosensitizer,” and “Indomethacin AND photodynamic therapy OR photosensitizer.” Articles were screened by title and abstract, followed by full-text review. Articles without original data and unrelated topics to IMC-PS in cancer therapy were excluded.

## Photodynamic Therapy and Its Mechanism

### Mechanism of photodynamic therapy

**Figure 1:**
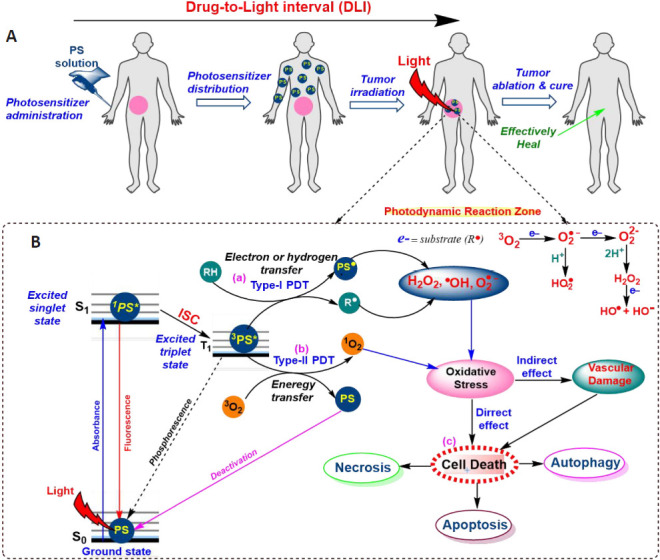
Principle and concept of PDT. (A) General schematic illustration of PDT procedure. (B) Schematic representation of photodynamic reaction mechanism. (a) Type I PDT reaction mechanism. (b) Type II PDT reaction mechanism. (c) Light-mediated type of cell death. Created with ChemDraw Professional 19.1. ·OH: Hydroxyl free radical; 1O_2_: singlet state of oxygen; 3O_2_: triplet state of oxygen; e-: electron; H^+^: hydrogen ion; H_2_O_2_: hydrogen peroxide; ISC: intersystem crossing; O_2_·^−^: superoxide anion free radical; PS: photosensitizer; PS*: singlet excited state of PS; S_0_: ground state; S_1_: excited singlet state; T1: relatively longer-lived stable triplet excited state PS.

PDT is a two-step medical treatment that involves the administration of PS to a tumor-bearing lesion or pathological tissue followed by local illumination of light of a specific wavelength, as illustrated in **Figure 1A**. The overall clinical efficacy of PDT is fundamentally governed by the spatial and temporal precise synergistic of three key components, namely a drug (PS), molecular oxygen, and light of a specific wavelength, as well as the delivery molecule or moiety appropriate to the target tissue, which collectively trigger the generation of ROS responsible for the destruction of tumor cells.^12^,^13^ First, the PS is administrated to the diseased tissue, either systemically through intravenous injection or topically, depending on the diseased tissue location and PS physicochemical properties. Following administration, the PS needs a period to accumulate in the diseased target tissue and achieve optimal concentration prior to light illumination. This interval of time between administration of a PS and applying the light source is called drug-light interval, which ranges from a few minutes to hours or days.

Once optimal PS accumulation has been achieved, the pathological tissue is illuminated with the light of a specific wavelength corresponding to the absorption maximum of the PS molecule.^4^,^14^ Upon illumination, the molecule (PS) is excited from the ground-state (S_0_) to a higher-energy first excited singlet state (S_1_, 1PS*). This excited singlet state PS is very unstable and loses its energy either by emitting fluorescence light or by internal conversion into heat. In some molecules, however, 1PS* may undergo a process known as “intersystem crossing (ISC)” to form a relatively longer-lived stable triplet excited state PS (T_1_, 3PS*) with parallel spins, which plays an important role in the generation of cytotoxic ROS. This excited triplet 3PS* molecule interacts with molecular oxygen (3O_2_, a triplet state at the ground state) through two major photochemical pathways: type I and II mechanisms, as shown in **Figure 1B**.

Type I mechanism: This type of photochemical process can occur whereby the 3PS* undergoes electron or proton transfer reaction with surrounding biomolecules to form radical anion or radical cation, respectively, which can further react with molecular oxygen to produce other ROS, such as superoxide anion (O_2_·^−^), hydrogen peroxide (H_2_O_2_), and hydroxyl radicals (·OH),^15^ as illustrated in **Figure 1Ba**.

Type II mechanism: This type of photochemical process can occur whereby the excess energy of 3PS* molecule can be transferred to molecular oxygen through collision to generate singlet oxygen (1O_2_), which is a highly cytotoxic ROS responsible for oxidative damage and cell death.^16^ This type of reaction mechanism is illustrated in **Figure 1Bb**. This further contributes as an alternative to enhance the cytotoxic effects.

In PDT applications, both type I and II photochemical processes can occur simultaneously while the ratio between these processes depends on the type of PS used, the substrate concentration and the local oxygen concentration within the targeted tissue microenvironment.^17^ Generally, designing of type II PSs is mechanically much easier than type I, and most clinical approved anticancer PDT agents are believed to work via the type II mechanism rather that type I reaction.

The response of cancer cells to PDT depends on several factors such as type of PS, drug concentration, photophysical properties and cellular localization of the PS, light dose applied, time of light illumination and oxygen concentration, as well as the cell type specific properties.^18^,^19^ While all of these influences the extent and mode of tumor cell death and contributes to the overall outcome of the treatment. This makes PDT a complicated treatment method to understand the molecular and cellular death mechanisms. PDT activates various types of cell deaths, including apoptosis, necrosis, and autophagy,^20^,^21^,^22^ as shown in **Figure 1Bc**. Additionally, PDT induces various cell death pathways, including pyroptosis and immunogenic cell death.^23^

### Cyclooxygenase-2 inhibitors and their role in cancer therapy

COX are key enzymes responsible for catalyzing the bis-oxygenation of arachidonic acid into a wide variety of PGs, which are potent ligand mediators involved in different inflammatory responses and pain.^24^,^25^ Two main COX isozymes have been identified: COX-1,^26^ which is responsible for hemostatic production of PGs and expressed in most normal tissues, and COX-2,^27^ which is an inducible enzyme that is expressed at a very low level or typically absent in normal cells and responsible for the production of pro-inflammatory PGs in inflammatory lesions. Furthermore, the COX-2 enzyme has an important role in the development of cancer because it inhibits apoptosis and promotes the process of angiogenesis. In contrast to COX-1, COX-2 is overexpressed in a wide range of malignant tumors, such as colorectal, breast, pancreatic, and lung cancer.^28^ Therefore, COX-2 has emerged as a new molecular target for pain and inflammation treatments.

Recently, studies have shown that COX-2 inhibitors significantly inhibit the PDT-induced local immunosuppression via inhibition of the COX-2 mediated pathway.^29^,^30^ Combining IMC and other COX-2 inhibitors with PDT offers a potential approach for reducing light-induced immunosuppression effects, thereby improving PDT efficacy or expanding its phototherapeutic applicability. Therefore, this underscores the urgent need to find an effective strategy to conjugate the IMC with near infrared (NIR)-absorbing PSs to achieve COX-2 targeted and immune-enhanced phototherapy.

## Indomethacin-Conjugated Photosensitizers

A lot of work has been done in recent years to develop conjugated PSs that target enzymes with favorable photophysical properties and biological activities.^31^ Enzyme-targeted conjugated PSs are PSs that have been chemically conjugated to ligands, which are moiety molecules that can bind to specific enzymes in cancer cells or within the tumor microenvironments.^32^ This conjugation strategy enhances tumor selectivity, minimizes off-target toxicity, and improves the overall efficacy of PDT by directing the PS specifically toward cancer cells. From a photophysical standpoint, the PDT agent should exhibit a strong absorption wavelength within the 600–800 nm range, where ROS generation and deep-tissue light penetration are optimal, as the photon energy beyond 800 nm is too low to efficiently generate ROS while below 600 nm limits tissue penetration.^33^

In this context, a wide range of organic dyes and their derivatives, such as cyanine, phenothiazine, phthalocyanine, boron dipyrromethene, and others, have demonstrated strong potential as enzyme-specific PSs for cancer treatment in PDT application. Thus, we focus on the recent development of IMC-conjugated PSs specifically designed to selectively target COX-2 overexpressing cancer cells.

### Heptamethine cyanine-based photosensitizer

Heptamethine cyanine (Cy7) dyes are a large group of cationic compounds characterized by two terminal nitrogen heterocyclic units between polymethine chain.^34^ Due to their hydrophobic cation, Cy7 dyes and their derivatives have been used as promising agents in fluorescence imaging, medical imaging, and phototherapy due to their broad absorption spectral ranges (600–900 nm) and high extinction coefficients. In Cy7 dyes and their derivatives, heavy atoms such as iodine are typically substituted at the meso-position into the aromatic ring structure, especially at the 5, 6, and 7 positions, to enhance ISC and boost singlet oxygen generation for improved PDT efficacy.^34^,^35^ This makes these dyes highly effective for bioimaging and phototherapy in the NIR region.

**Figure 2:**
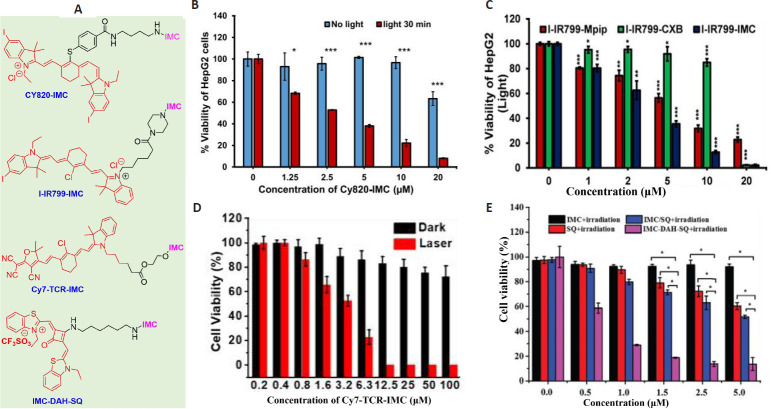
IMC-Cy7-based conjugated PSs. (A) Molecular structure of various IMC-Cy7 conjugated PS. (B) Cytotoxicity of Cy820-IMC. Reprinted from Siriwibool et al.^36^ Copyright 2022 Elsevier Inc. (C) Cytotoxicity of I-IR799-IMC. Reprinted from Wangngae et al.^11^ Copyright 2022 Wiley‐VCH GmbH. (D) Cytotoxicity of Cy7-TCR-IMC. Reprinted from Mu et al.^37^ Copyright 2021 American Chemical Society. (E) Cytotoxicity of IMC-DAH-SQ. Reprinted from Wang et al.^38^ Copyright 2021 Royal Society of Chemistry. Cy7-TCR-IMC: Indomethacin-conjugated heptamethine cyanine Cy7 tricyanofuran photosensitizer; CY820-IMC: Indomethacin-conjugated iodinated heptamethine cyanine Cy820 photosensitizer; HepG2 cell: hepatocellular cancer cell; I-IR799-CXB: celecoxib-conjugated iodinated heptamethine cyanine IR799 photosensitizer; I-IR799-IMC: indomethacin-conjugated iodinated heptamethine cyanine IR799 photosensitizer; I-IR799-Mpip: piperazine-conjugated iodinated heptamethine cyanine IR799 photosensitizer; IMC: indomethacin; IMC-DAH-SQ: squaraine dye conjugated indomethacin; PS: photosensitizer; SQ: squaraine.

Recently, a few IMC-conjugated Cy7-based PSs have been developed by incorporating alkyl linkers between the IMC moiety and Cy7 derivatives for the treatment of COX-2 overexpressing cancer cells, as shown in **Figure 2A**. For example, Kamkaew’s team developed COX-2 specific NIR fluorescent sensitizers, Cy820-IMC, by conjugating the IMC moiety into diiodinated heptamethine cyanine (Cy820) dye through a tetradiamine link.^36^ This approach targets COX-2 enzymes and eradicates tumor cells using the light-induced cytotoxicity effect. The results showed that Cy820-IMC accumulated in many COX-2 overexpressing cancer cells, indicating the internalization via organic anion transporting polypeptides and IMC targeting ligand in response to Cy820-IMC binding. As shown in **Figure 2B**, the cancer cell viability decreased dramatically in a dose-dependent manner as the concentrations of Cy820-IMC increased from 1 to 20 μM upon 780 nm illumination, while the cells maintained high viability in the dark state at concentrations up to 10 μM. However, a significant decrease in cell viability was observed when the dose was increased to 20 μM, indicating cytotoxicity at higher concentrations in the absence of light.

From the same group, another COX-2-specific NIR fluorescent sensitizer, I-IR799-IMC, is reported for imaging COX-2 enzymes and eradicating tumor cells using the same target ligand, IMC.^11^ In their design, the authors conjugate the I-IR799 NIR PS to IMC via a flexible linker. The findings showed that I-IR799-IMC exhibited the lowest half maximal effective concentration value (EC50; 3.03 μM) compared to the control probes under an 805 nm light illumination for 15 minutes, especially the cell viability was significantly reduced in cancer cells compared to I-IR799-CXB (a celecoxib-based derivative; **Figure 2C**). However, these probes still pronounced dark cytotoxicity at concentrations higher than 20 μM, in addition to significant light-induced cytotoxicity in normal cells. These safety issues hinder their potential application in clinical settings and require further optimization.

Using a similar approach, a Cy7-based IMC photothermal agent, Cy7-TCF-IMC, that self-assembles into supramolecular nanosaucers, has been developed for dual active targeting effects in image-guided photothermal cancer therapy.^37^ As shown in **Figure 2D**, the photothermal effect of Cy7-TCF-IMC on HeLa cancer cells resulted in a decrease in cell viability as concentration increased upon light irradiation. For instance, almost no living cells survived above 4.0 μM. In the absence of light, for comparison, Cy7-TCF-IMC didn’t show a dark cytotoxicity effect on cells even when treated with 25.0 μM (> 90%). These findings demonstrate that the self-assembling nature of Cy7-TCF-IMC nanosaucers affords excellent photothermal effects and preferential accumulation in COX-2 overexpressing tumor cells with a desirable biocompatibility profile.

In another study, squaraine (SQ) incorporated cyanine-based nanoscale PS, named IMC-DAH-SQ, has been developed by conjugating IMC moiety and SQ derivatives through a short flexible chain.^38^ IMC-DAH-SQ is a dual-targeting phototheranostic nanoagent that incorporates structure-inherent targeting and a COX-2 binding moiety, enabling tumor-selective phototherapy and OFF-ON imaging. Unlike the conventional COX-2 specific PSs, its amphiphilic nature allows self-assembly into a nanoprobe with a low background fluorescence in normal cells. Upon accumulation in COX-2 expressing tumor cells, the nanoprobe disassembles into monomeric units, leading to a turn-on NIR fluorescent signal for precise image-guided PDT. As shown in **Figure 2E**, upon light irradiation (25 mM/cm^2^), the viability of tumor cells pretreated with IMC-DAH-SQ decreased significantly with increasing concentration, with 0.59 μM half-maximal inhibitor concentration. All these findings demonstrate that IMC-DAH-SQ confers the nanoprobe with high tumor selectivity and holds significant promise for imaging-guided antitumor phototherapy.

### Phenothiazine and Nile Blue-based photosensitizer

Research has shown that heavy atom substitution of commercially known Nile Blue dye with sulfur and selenium atom yielded several series of NIR PS analogues with improved ISC efficiency, high photostability, high ROS generation, and strong light absorption in the NIR region (λ_ab_ = 650–680 nm).^39^,^40^,^41^

**Figure 3:**
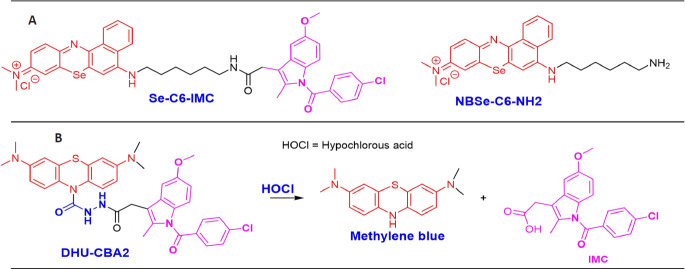
IMC-phenoxazine-based conjugated PSs. (A) Molecular structure of Se-C6-IMC and NBSe-C6-NH_2_. (B) Hypochlorous acid-responsive reaction of DHU-CBA2 and its products. Created with ChemDraw Professional 19.1. DHU-CBA2: Indomethacin-conjugated methylene blue based photosensitizer; NBSe-C6-NH_2_: indomethacin free benzo[a]phenoselenazine based photosensitizer; PS: photosensitizer; Se-C6-IMC: indomethacin-conjugated benzo[a]phenoselenazine based photosensitizer.

The introduction of the sulfur or selenium atom at the 10-position of the Nile blue scaffold results in decreased fluorescence intensity and enhanced ROS generation due to the heavy atom effect. This makes it ideal for deep-tissue bioimaging and photodynamic activity in biological applications. Based on a Nile blue-based scaffold, a novel NIR PS, Se-C6-IMC, is developed by conjugating benzo[a]phenoselenium-based dye with IMC moiety via linear hexanediamine by our group.^9^ To understand the tumor-targeting potency, an IMC-lacking PS, NBSe-C6-NH_2_, was prepared as control (**Figure 3**). Upon NIR light irradiation at 660 nm, Se-C6-IMC exhibited a dramatic time-dependent decreasing of 1,3-diphenylisobenzofuran absorbance at 415 nm in the CH_2_Cl_2_ but not in the Tris-HCl buffer solution, showing more efficient ROs generation. Similarly, upon excitation of 631 nm laser, Se-C6-IMC showed strong red fluorescence emission in the MCF-7 cells, while a negligible fluorescence was observed in the COX-2-negatively expressed COS-7 normal cells. This further confirms the aggregation inhibition in the cancer cells. The intracellular ROS generation investigation further demonstrated that a negligible green fluorescence was observed in cells treated without light, NBSe-C6-NH_2_ or Se-C6-IMC alone. In contrast, a bright green fluorescent was observed in cells treated with both PSs upon 660 nm NIR laser irradiation, indicating that the high-level fluorescence observed in the cells demonstrates high production of ROS in COX-2-expressing cancer cells. Therefore, these results clearly confirmed that the production of 1O_2_ in MCF-7 cells is, in fact, responsible for the apoptosis of tumor cells.

In *in vitro* cytotoxicity study, the author further examined Se-C6-IMC-iduced cell death using the MTT assay, and exhibited a negligible dark toxicity against both MCF-7 and COS-7 cells, suggesting its good biocompatibility in biological systems. It also showed a significant inhibition of MCF-7 cells in dose-dependent manner as light-dose increased from 3.6 to 14.4 J/cm^2^ over a wide range of concentration (0.02 to 1.25 µM), indicating that Se-C6-IMC selectively ablates COX-2-overexpressed cancer cells than control ones. In addition, Se-C6-IMC showed the highest Golgi apparatus localization, leading to more effective protection against cellular damage. Thus, Se-C6-IMC might have a great COX-2-specific NIR PS for improving the PDT efficiency in future. Similarly, Wang et al.^42^ synthesized a hypochlorous acid (HOCl)-responsive prodrug (DHU-CBA2) by grafting PS methylene blue and IMC using acyl hydrazine as the tumor-specific response group, as illustrated in its molecular structure in **Figure 3B**. Using this prodrug, the authors investigated the combined effect of PDT and COX-2 inhibitor in lung cancer and spinal metastasis. DHU-CBA2 responded selectively to HOCl and simultaneously released methylene blue and IMC in lung tumor cells and in the tumor microenvironment of metastasis, showing good dual photodynamic and pharmacological action in both *in vitro* and *in vivo*. Furthermore, activated DHU-CBA2 exhibited that IMC could effectively inhibit PDT-induced PGE2 expression upon light irradiation. Thus, this study introduces an innovative dual-functional autoboost strategy for releasing methylene blue and IMC in response to HOCl, while its PDT application presents limitations in deep-seated and visceral tumor treatments.

### Porphyrin and phthalocyanine-based photosensitizer

**Figure 4:**
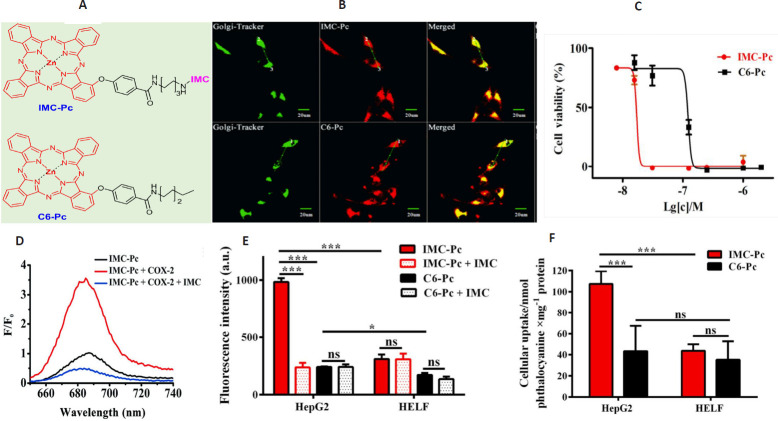
IMC-phthalocyanine-based PS. (A) Molecular structure of IMC-Pc and its parent PS. (B) Intracellular co-localization of IMC-Pc (1 μM, 24 hours) and C6-Pc (1 μM, 24 hours) with Golgi-Tracker Green (2 μM, 60 minutes) staining in HepG2 cells. (C) Photocytotoxicity of IMC-Pc (red) and C6-Pc (black) towards (670 nm, 12.5 mW/cm^2^, 1.5 J/cm^2^). (D) Fluorescence spectra of IMC-Pc (2 μM) and C6-Pc (2 μM) in 100 mM Tris-HCl buffer (pH = 8.0) with COX-2 (0 and 150 nM) and IMC (0 and 20 μM). (E) Quantified average fluorescence intensity of the confocal images. (F) Cellular uptake of HepG2 and HELF cells after incubation with 0.25 μM IMC-Pc and C6-Pc. Reprinted from Huang et al.^45^ Copyright 2021 Elsevier Ltd. C6-Pc: IMC-Pc parent compound; COX-2: cyclooxygenase 2; HELE cell: human lung fibroblasts normal cell; HepG2 cell: hepatocellular cancer cell; IMC: indomethacin; IMC-Pc: indomethacin-conjugated zinc phthalocyanine photosensitizer; PS: photosensitizer.

Recently, scientific research has shown that porphyrin, chlorin, and phthalocyanine derivatives are widely used in the development of modern PSs for cancer treatments because of their strong light absorption, efficient ROS generation, and aggregation in aqueous solution.^43^,^44^ For instance, a novel PS is developed by introducing the COX-2 binding group IMC to zinc phthalocyanine derivatives to overcome potent aggregation and poor tumor targeting of conventional PSs.^45^ The proposed PS (IMC-Pc) and its parent compound (C6-Pc) (**Figure 4A**) were validated using fluorescence measurement and docking calculations. Compared to C6-Pc, the IMC moiety thus helped IMC-PC target only COX-2 highly expressing tumor cells and then accumulate in the Golgi apparatus (*r* = 0.92, as illustrated in **Figure 4B**. The *in vitro* study showed that IMC-Pc displayed ultra-efficient and COX-2 targeted photocytotoxicity in HePG2 cancer cells (**Figure 4C**), while still maintaining negligible photocytotoxicity against HELF normal cells. Thus, IMC-Pc showed a 7.2-fold lower IC50 value (17.1 nM) against HepG2 cancer cells compared to control compound C6-Pc, indicating significantly higher ROS generation. As shown in **Figure 4D**,^45^ IMC-Pc further exhibited little fluorescence intensity in Tri-HCl buffer and pretreated IMC, while its fluorescence intensity increased at 687 nm after incubating with 150 nM COX-2 protein. Similarly, significant intracellular fluorescence intensity of IMC-Pc is observed in HepG2 cells compared to HELF cells and HepG2-pretreated with free IMC (**Figure 4E**). Thus, these results demonstrate the role of the IMC moiety in reducing the aggregation and improving the fluorescence intensity of IMC-Pc compared to its parent control compound. The introduction of the IMC moiety in ZnPc further improved cellular uptake of IMC-Pc in cancer cells (**Figure 4F**). Overall, these findings demonstrated that IMC-Pc is a potent PS for improving treatment efficacy due to its “three-in-one” COX-2 driven dual targeting and aggregation inhibition. In another study, polphyrin- and chlorin-IMC conjugates, named P2-InD and C2-Ind, respectively, have been developed for treating COX-2 expressing cancer cells with PDT effect.^46^ However, dark cytotoxicity and low photocytotoxicity of the conjugates due to aggregation formation were still major concerns.

### Other photosensitizer derivatives

**Figure 5:**
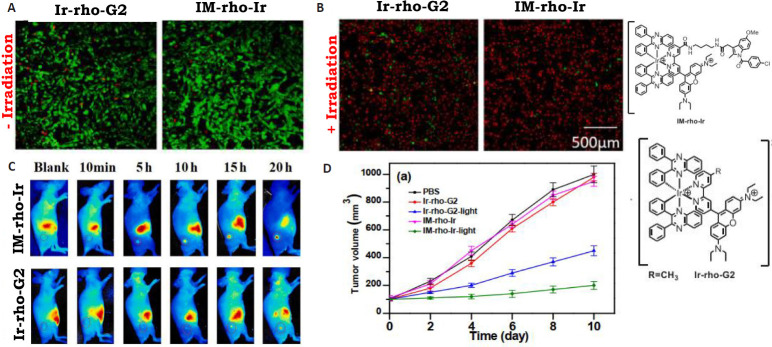
IMC-conjugated rhodamine-iridium (III) based PS. (A) Dark cytotoxicity analysis. Viable cells were stained green with Calcein-AM. (B) light-induced cytotoxicity after irradiation with lamp (11 W/cm^2^) for 20 minutes. Dead cells were stained red with PI. (C) *In vivo* fluorescence images of MCF-7 tumor-bearing nude mice ranging from 0 to 20 hours. (D) *In vivo* tumor volume changes during PDT. Reprinted from Liu et al.^49^ Copyright 2021 Elsevier Inc. IM-rho-Ir: A novel conjugate rhodamine-iridium (III) complex; IR-rho-G2: indomethacin free rhodamine-iridium photosensitizer; MCF-7 cell: human breast cancer cell; PBS: phosphate buffered solution; PDT: Photodynamic therapy; PI: propidium iodide; PS: photosensitizer.

Recent research has demonstrated cyclometalated iridium (III) complexes as promising candidates for PDT applications and metallo-anticancer applications, owing to their unique excellent targeting ability, structural tenability, and favorable optical properties.^47^,^48^ For example, Liu et al.^49^ developed a novel conjugate rhodamine-iridium (III) complex, IM-rho-Ir, by covalently attaching the IMC moiety to the parent Ir-rho-G2 compound to enhance the COX-2 expressed cancer cell selectivity (**Figure 5A**). In their design, Ir-rho-G2 function as the PS unit capable of generating 1O_2_ upon light irradiation to induce cell death, and IMC is a directing unit to bind the COX-2 enzyme, which is overexpressed in many cancer cells. Compared to the parent compound, IM-rho-Ir exhibited weaker fluorescence (Φ = 0.007) and 1O_2_ generation (Φ_Δ_ = 70%). These findings confirm that the Ir (III)-conjugated system significantly enhances PDT activity through efficient 1O_2_ generations. Furthermore, both IM-rho-Ir and Ir-rho-G2 exhibited low intercellular ROS generation in MCF-7 cells without the light irradiation (**Figure 5B**), suggesting their low dark toxicity. In contrast, upon light irradiation with a lamp (11 W/cm^2^) for 20 minutes, both showed potent photodynamic cytotoxicity with less than 1% viable cells (**Figure 5C**), as indicated by green calcein-AM and red propidium iodide fluorescence. The tumor selectivity of both IM-rho-Ir and Ir-rho-G2 has been further evaluated in MCF-7 bearing Female nude mice by monitoring the fluorescence tumor accumulation from 0 to 20 hours after intravenous injection. As shown in **Figure 5D**, fluorescence signals were observed in both groups, even at 20 hours post-injections, clearly indicating higher uptake efficiency at the tumor sites. Compared to the control group, IM-rho-Ir showed more PDT effect upon light irradiation and was more potent than rho-Ir-G2 (**Figure 5E**), indicating remarkable tumor growth suppression, even on the 10^th^ day post-treatment. However, the shorter excitation wavelength of IM-rho-Ir in the visible region hinders its potential for broader clinical application in cancer treatment.

In another study, Wang et al.^10^ reported an aggregation-induced emission-based PS, named TTPI, for cancer-selective imaging and photodynamic-induced apoptosis in cancer cells. They designed a push-pull cationic PS-based D-π-A system to synthesize triphyenylphosphine derivative (TPP) by using triphenylamine as a backbone and a thiophene moiety as a π-spacer. This allowed TPP to exhibit improved ISC efficiency and extended the emission wavelength to the NIR region. To improve the tumor selectivity, the author synthesized TPP1 by linking the IMC moiety to TPP. Upon light irradiation, TPP1 demonstrated the ability to generate both type I and II ROS photochemical pathways in A549 cells, highlighting its strong potential as a type I and II PS for Golgi apparatus-specific treatment (Pearson’s *r* = 0.960). Furthermore, the *in vitro* and *in vivo* experimental results demonstrate that “TPP1+light” exhibited significant photodynamic anticancer activity, with a tumor inhibition rate reaching 64.32%. These findings highlight the strong potential of TPP1 as an effective type I and II PS for PDT application in cancer treatments. Similar to the IM-rho-Ir, the shorter excitation wavelength of TTP1 (λ_max_ = 485 nm) in the visible region limits its potential for further application in clinical settings.

## Discussion

A recent study suggest that the effectiveness of light-induced therapies is affected by the local PDT-mediated immunosuppressive occurrence through a COX-2 mediated pathway, which acts as a negative regulator of immune responses.^29^ This limitation can be mitigated by inhibiting the COX-2/PGE2 signaling axis. IMC and other COX-2 inhibitors have shown promise in inhibiting PGE2 production, thereby making cancer cells more sensitive to other treatments. Thus, targeting the COX-2 enzyme using IMC with PDT offers a potential approach for reducing light-induced immunosuppression and could enhance PDT efficacy or expand PDT indications at the diseased tissues. In this review, we highlight the multifunctional therapeutic potential of IMC-PS conjugates in cancer research that integrate the COX-2 inhibitory activity of IMC with the varieties of photosensitizing agents.

**Figure 6:**
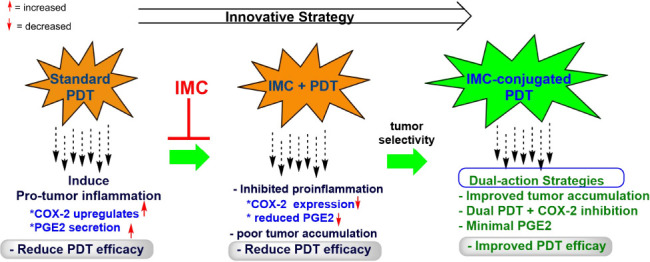
Innovative strategies for IMC-conjugated photosensitizers. Created with ChemDraw Professional 19.1. COX-2: Cyclooxygenase 2; IMC: indomethacin; PDT: photodynamic therapy; PGE2: prostaglandin E2.

As illustrated in **Figure 6**, the efficacy of conventional PDT is affected by the induction of COX-2/PGE2-mediated protumorigenic inflammation. IMC, including other COX-2 inhibitors, can inhibit PDT-mediated resistance by blocking the COX-2/PGE2 pathways.^50^ The combination of IMC with PDT can address this limitation by suppressing protumor inflammatory signaling.^51^ Notably, IMC-conjugated PSs offer an innovative dual-action strategy, resulting in simultaneous suppression of protumor inflammation responses and enhancing tumor accumulation and increase the overall efficacy of the PDT.

These IMC-conjugated PDT agents provide a feasible scheme for the COX-2 targeted treatment of tumors and have attracted increasing attention in recent studies. The photophysical properties of these PDT agents are summarized in **Table 1**. These molecules have been rationally engineered by chemically linking IMC anticancer drug to various PS chromophores, including Cy7s, phthalocyanines, phenothiazines, benzo[a]phenoselenazines, porphyrins, and Ir (III) complex derivatives. These alkyl linkers act as flexible molecular spacers that not only modulate the steric and electronic interactions between the IMC and PS units but also influence the overall amphiphilicity, solubility, photophysical parameters, and cellular uptake and localization of these conjugates. Notably, these conjugates with longer chains, such as IMC-Pc, Se-C6-IMC, and Cy7 conjugates, demonstrate enhanced spatial flexibility and exhibit higher binding affinity and selectivity of the IMC moiety toward the COX-2 active site, thereby impacting the ROS generation upon light irradiation.^9^,^45^ Such structural optimization is essential to enhance selective accumulation in COX-2 overexpressing inflamed or tumor cells/tissues while maintaining its photophysical properties. Thus, linker scaffold optimization is a key design strategy to balance COX-2-targeted binding sites and efficient photodynamic response.

**Table 1:** Comparative photophysical properties of IMC-conjugated PS

Type of IMC-conjugated PS	Absorption (λ_max_)	Singlet oxygen quantum yield	Type of PDT mechanism	Inhibition and light dose	Reference
Cy820-IMC	822 nm in PBS	0.12 in PBS	Type II	IC_50_ = 2.8 μΜ 2.37 mW/cm^2^, 780 nm lamp, 30 min	36
I-IR799-IMC	798 nm in PBS	0.12 in PBS	Type II	EC_50_ = 3.03 μΜ 18.0 mW/cm^2^, 805 nm lamp, 15 min	11
Cy7-TCF-IMC	790 nm in H_2_O	-	Type II	EC_50_ = 3.2 μΜ 1.0 W/cm^2^, 805 nm lamp, 10 min	37
IMC-DAH-SQ	654 nm in H_2_O	0.035 in PBS	Type II	IC_50_ = 0.59 μΜ 25.0 mW/cm^2^, 630 nm lamp, 12.5 min	38
IMC-Pc	674 nm in DMF	0.59 in DMF	Type II	IC_50_ = 0.17 μΜ 12.5 mW/cm^2^, 670 nm lamp, 10 min	45
Se-C6-IMC	660 nm in PBS	0.74 in CH_2_Cl_2_	Type I	IC_50_ = 0.04 μM 14.4 J/cm^2^	9
IM-rho-Ir	550 nm in CH_3_CN	0.70 in CH_3_CN	Type I	IC_50_ = 1.80 μM 11 W/cm^2^, 20 min	49

CH_2_Cl_2_: Dichloromethane; CH_3_CN: acetonitrile; Cy7-TCR-IMC: Indomethacin-conjugated heptamethine cyanine Cy7 tricyanofuran photosensitizer; CY820-IMC: indomethacin-conjugated iodinated heptamethine cyanine Cy820 photosensitizer; DMF: N,N-Dimethylformamide; EC_50_: median effective concentration; H_2_O_2_: hydrogen peroxide; I-IR799-IMC: indomethacin-conjugated iodinated heptamethine cyanine IR799 photosensitizer; IC_50_: median inhibition concentration; IMC: indomethacin; IMC-DAH-SQ: squaraine dye conjugated indomethacin; IMC-Pc: indomethacin-conjugated zinc phthalocyanine photosensitizer; IM-rho-Ir: a novel conjugate rhodamine-iridium (III) complex; PBS: phosphate buffered solution; PS: photosensitizer; Se-C6-IMC: indomethacin-conjugated benzo[a]phenoselenazine based photosensitizer.

In IMC-PSs design, the choice and length of linkers critically influence the specific binding to a COX-2 active site as well as solubility, self-aggregation and cellular uptake. Flexible linkers, such as C6 or higher alkyl chain units, as demonstrated in IMC-DAH-SQ,^38^ Cy7-TCR-IMC,^37^ and Se-C6-IMC,^9^ can enhance cellular uptake and reduce self-aggregation in COX-2 overexpressed cancer cells. Aromatic with short alkyl linker, like IMC-PC and Cy820-IMC,^36^ provides precise spatial orientation between IMC and PC, enhancing binding affinity to COX-2 overexpressing tumor cells. Cleavable linker, like DHU-CBA2,^42^ responsive to hypochlorous acid, enables selective release of the PS and enhances tumor targeting. Systematic variation of linker length, rigidity, and functional groups can therefore markedly influence Golgi-apparatus localization, ROS generation efficiency, and overall PDT efficacy of IMC-PSs conjugates.

Collectively, these findings highlight the promising photodynamic performance of the IMC-PS conjugates *in vitro*, particularly their preferential accumulation in COX-2 overexpressing tumor cells, which enables more selective and effective therapeutic interventions. The conjugation of IMC to PSs not only enhances cellular accumulation but also improves PDT effectiveness relative to their parent PS by mitigating the immunosuppression of tumor through inhibition of the COX-2/PGE2 pathway.^42^ These dual functions, light-induced ROS generation and COX-2 inhibition, provide synergistic anticancer effects. Despite these advantages, several safety-related issues remain to be resolved before translation to clinical applications, including dark toxicity, self-aggregation tendencies, and suboptimal photophysical properties, which could compromise tumor selectivity and increase side effects. Addressing these integrated challenges through rational molecular design of PSs, linker optimization, and incorporation of COX-2 inhibitors will be fundamental in enhancing the translational potential of COX-2 targeted PSs.

## Conclusion and Outlook

PDT-induced treatments are affected by COX-2/PGE2 signal axis, which affects immune response. IMC and other COX-2 inhibitors can inhibit PGE2 production, making cancer cells more sensitive to other therapies. IMC-PS conjugates offer a feasible scheme for COX-2 targeted PDT. Despite significant advancements in the development of IMC-PS conjugates for COX-2 targeted PDT application, several key challenges remain to be addressed. These include limitations associated with dark cytotoxicity, self-aggregation tendencies, suboptimal photophysical properties, and linker design, which influences phototherapeutic efficacy, safety, and tumor selectivity. Moreover, these conjugates have been evaluated only *in vitro*, without full *in vivo* validations. This lack of *in vivo* investigation, combined with the observed safety issues and suboptimal photophysical properties, significantly limits the potential application of these IMC-PS conjugates in clinical settings. In the following sections, we outline strategic approaches to mitigate these limitations.

(a) Suboptimal photophysical properties: Many of these IMC-PS conjugates absorb light maximally in the visible range (< 600 nm), which makes it harder for them to penetrate deep tissue and raises the risk of dark toxicity and off-target phototoxicity. To fix this, the red-shifting IMC-PS absorption into 600–800 nm therapeutic window can be achieved through several complementary structural modification approaches: (i) extending π-conjugation via β-/meso-substitution or fused aromatic rings lower the energy HOMO-LUMO gap, as demonstrated in porphyrins, cyanines, and cyclometalated Ir(III) complexes; (ii) push-pull substitution with electron-withdrawing and electro-donating groups across the PS further polarizes the π-conjugation systems, enhancing bathochromic shifts; (iii) metalation or incorporation of heavy atoms, such as Zn and Se, tunes HOMO-LUMO electronic energy gap, enhances ISC effects as demonstrated in IMC-PC^45^ and Se-C6-IMC,^9^ respectively, and (iv) incorporation of bulky substituents, such as liposomal, polymeric materials, or PEGylation, maintains monomeric in biological microenvironments and preserves the desired red-shifted photo-optical properties, which is collectively improving tissue penetration, ROS generation efficiency, and safety. Furthermore, some of them are not very effective and demonstrate relatively low PDT efficiency, requiring prolonged light exposure and higher fluence rates to achieve phototherapeutic efficacy. This prolonged light illumination not only increases treatment time but also increases the risk of off-target phototoxicity, patient discomfort, and thermal damage to adjacent healthy tissues. This can be addressed by enhancing the ISC efficiency of the PS, thereby facilitating more effective population of the triplet excited state and improving ROS generation, thereby enabling effective PDT at shorter light irradiation periods and lower light dose.

(b) Dark toxicity and off-target cytotoxicity: Some IMC-PS conjugates, such as Cy7-derivatives, highlight significant cytotoxicity even in the absence of light illumination, particularly at concentrations exceeding 20 μM, which raises safety concerns. To address this issue, antioxidants like polyphenols are employed as free ROS scavengers to protect health cells and tissues from the adverse effect of PDT-induced oxidative damage.^52^

(c) Molecular oxygen availability: The presence of sufficient molecular oxygen is crucial for efficient production of ROS, particularly singlet oxygen, which is the primary cytotoxic agent responsible for inducing cell death in PDT. The photodynamic efficacy of these IMC-PS conjugates is inherently dependent on the sufficient presence of molecular oxygen at the treatment site. As a result, these conjugates limit their therapeutic activities under hypoxic conditions, which are common phenomena observed in solid tumors and poorly oxygenated tumor microenvironments. To overcome these challenges, several strategies have been explored.^53^,^54^

(d) Self-aggregation in tumor cells: Some IMC-PS conjugates like porphyrins and chlorin derivatives, exhibit self-aggregation in both aqueous solution and tumor cells, which leads to reduced ROS generation, fluorescence quenching, and decreased PDT efficacy. To mitigate this issue, stabilizers, such as liposomes, polymeric carriers, and cyclodextrins, are incorporated to improve the physicochemical stability of these PSs and reduce self-aggregation within biological environments.^55^

Collectively, these findings underscore the urgent need for further molecular optimization of IMC-PS photoagents and full *in vivo* investigation to improve their photostability, biocompatibility, phototherapeutic performance, and translational potential, as well as to minimize self-aggregation. The development of next-generation PDT agents should aim for high photodynamic performance, minimal off-target toxicity, enhanced biocompatibility and tumor-selective accumulation. These approaches hold promise for achieving safe, selective, and clinically effective PDT.

## Data Availability

*Data will be made available on request.*
